# New Regions With Molecular Alterations in a Rare Case of Insulinomatosis: Case Report With Literature Review

**DOI:** 10.3389/fendo.2021.760154

**Published:** 2021-10-19

**Authors:** Kirill Anoshkin, Ivan Vasilyev, Kristina Karandasheva, Mikhail Shugay, Valeriya Kudryavtseva, Alexey Egorov, Larisa Gurevich, Anna Mironova, Alexey Serikov, Sergei Kutsev, Vladimir Strelnikov

**Affiliations:** ^1^ Laboratory of Epigenetics, Research Centre for Medical Genetics, Moscow, Russia; ^2^ I.M. Sechenov First Moscow State Medical University, Moscow, Russia; ^3^ Pirogov Russian National Research Medical University, Moscow, Russia; ^4^ Shemyakin-Ovchinnikov Institute of Bioorganic Chemistry, Russian Academy of Science, Moscow, Russia; ^5^ Privolzhsky Research Medical University, Nizhny Novgorod, Russia; ^6^ Morphological Department of Oncology, M.F. Vladimirsky Moscow Regional Research and Clinical Institute, Moscow, Russia

**Keywords:** neuroendocrine tumors, FOXL2, IRS2, CEBPA, copy number variation, insulinomatosis

## Abstract

Insulinomatosis is characterized by monohormonality of multiple macro-tumors and micro-tumors that arise synchronously and metachronously in all regions of the pancreas, and often recurring hypoglycemia. One of the main characteristics of insulinomatosis is the presence of insulin-expressing monohormonal endocrine cell clusters that are exclusively composed of proliferating insulin-positive cells, are less than 1 mm in size, and show solid islet-like structure. It is presumed that insulinomatosis affects the entire population of β-cells. With regards to molecular genetics, this phenomenon is not related to mutation in *MEN1* gene and is more similar to sporadic benign insulinomas, however, at the moment molecular genetics of this disease remains poorly investigated. NGS sequencing was performed with a panel of 409 cancer-related genes. Results of sequencing were analyzed by bioinformatic algorithms for detecting point mutations and copy number variations. DNA copy number variations were detected that harbor a large number of genes in insulinoma and fewer genes in micro-tumors. qPCR was used to confirm copy number variations at *ATRX*, *FOXL2*, *IRS2* and *CEBPA* genes. Copy number alterations involving *FOXL2*, *IRS2*, *CEBPA* and *ATRX* genes were observed in insulinoma as well as in micro-tumors samples, suggesting that alterations of these genes may promote malignization in the β-cells population.

## Introduction

Multiple insulinomas are most common in MEN1 syndrome, however, several studies show that they can also occur sporadically, albeit very rarely, and are referred to insulinomatosis (1–3). Term “insulinomatosis” was introduced and described by Anlauf et al. (1). It is characterized by monohormonality of multiple macrotumors and microtumors that arise synchronously and metachronously in all regions of the pancreas, rare of metastases, and often recurring hypoglycemia. In addition, one of the main characteristics of insulinomatosis is the presence of insulin-expressing monohormonal endocrine cell clusters that are exclusively composed of proliferating insulin-positive cells, are less than 1 mm in size, and show solid islet-like structure. Authors suggested that insulinomatosis affects the entire population of β-cells (1, 2). With regards to molecular genetics, this phenomenon is not related to mutation in *MEN1* gene and is more similar to sporadic benign insulinomas, however, large-scale molecular genetic studies on this disease have not yet been carried out (1). Here we present a very rare case of insulinomatosis in a patient with hypoglycemia syndrome and without known hereditary syndromes.

## Case Presentation

A male in his 60s with clinical signs of hypoglycemia was admitted to the surgical department of Sechenov University. The patient signed informed agreement to undergo diagnostic procedures and treatment, as well as to participate in the study, and for the presentation of clinical and molecular data in scientific and medical literature. This case report was approved by the local Ethics Committee at the Research Centre for Medical Genetics, Moscow, Russia.

The history of the disease is about 6 years with fasting serum glucose 2.7-3.7 mmol/l compensated by sugar intake. The 72-hour fasting test was negative. The past medical history is significant for combination treatment for laryngeal carcinoma T2N0M0, stage II. Currently there are no signs of recurrence. CT scan of the abdomen shows heterogeneous hypervascular soft tissue mass up to 26 mm suspicious for neuroendocrine tumor (NET), located within the tail of the pancreas, with a well-defined cystic component attached to the splenic artery and vein without invasion. Hypervascular lesions of up to 5 mm without clear borders are detected within the surrounding parenchyma of the pancreatic tail.

### Treatment

Based on the obtained diagnostic data, the multidisciplinary board was held, implying the participation of surgical oncologist, medical oncologist, pathologist and radiation oncologist. Taking into account tumor size more than 2 cm and hypoglycemia syndrome radical pancreatic resection was planned. A distal spleen-preserving pancreatectomy was performed.

### Outcome

Taking into account multiple microadenomas in the pancreatic parenchyma the recurrence rate of the hyperinsulinemic hypoglycemia is relatively high. Thus, patient is examined every 3-6 month. In our case there are no signs of hypoglycemia and no pancreatic tumors on imaging during 30 months of follow-up by CT and PET-CT scanning.

### Microscopy and Immunohistochemistry

**Figure 1:**
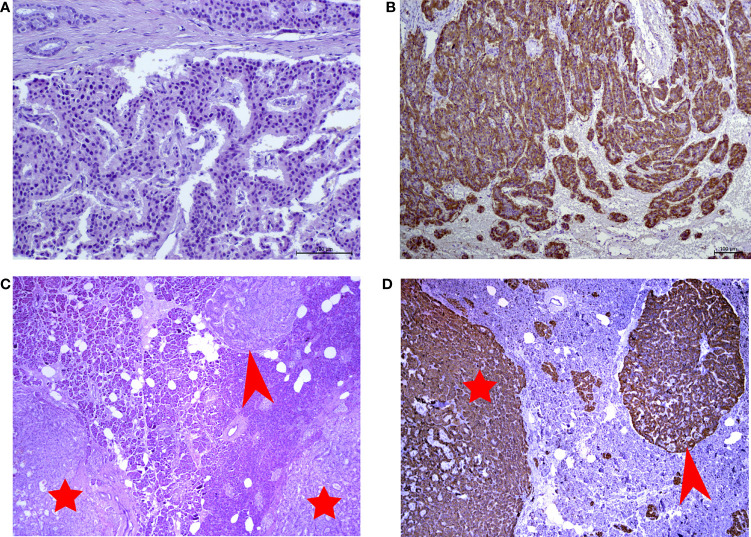
**(A)** Hematoxylin and eosin (H&E)-stained slide of insulinoma (x250 magnification). **(B)** Immunohistochemical reaction with antibodies to insulin in insulinoma (x125 magnification). **(C)** Hematoxylin and eosin (H&E)-stained slide of microtumors (x10 magnification). **(D)** Immunohistochemical reaction with antibodiesto insulin in microadenoma (x10 magnification). Red arrows point to insulin-producing microadenomas. Red stars indicate the microtumors.

Microscopic examination showed well differentiated neuroendocrine tumor with 2 mitoses and no necrosis in 10 HPF. There are multiple large microadenomas in the surrounding pancreatic tissue. Tumor cells are positive for synaptophysin and chromogranin A, as well as for insulin (**Figures 1A, B**). There is no expression of glucagon, somatostatin and pancreatic polypeptide. Ki-67 is less than 1.5%, Grade 1. The cells of microadenomas express insulin and are negative for glucagon and somatostatin (**Figures 1C, D**).

## Materials and Methods

### DNA Extraction

Macrodissection of tumors was performed under guidance of pathologist. DNA was extracted from four formalin-fixed, paraffin-embedded (FFPE) tumor tissues by using GeneRead DNA FFPE kit (Qiagen, Germany), and from whole peripheral blood by using standard phenol-chloroform extraction protocol. One tumor sample was insulinoma, three others were small proliferative insulin-expressing monohormonal endocrine cell clusters (IMECCs).

### NGS Sequencing

Next generation sequencing (NGS) was performed by using Ion AmpliSeq targeted amplification technology with AmpliSeq Comprehensive Cancer Panel (Thermo Fisher Scientific, United States) with exon coverage of 409 oncogenes and tumor suppressor genes.

### Bioinformatic Analysis

The bioinformatic workflow for sequencing data analysis was based on Torrent Suite software (version 5.10.1). Annotation was performed by ANNOVAR (4). CNVs were called using CNVpanelizer R package (version 1.22.0) ran with default parameters (5). Putative CNV status is assigned when there is a significant difference between observed copy number and the one expected from the bootstrapped distribution. A reliable CNV is called when the upper bound of the copy number ratio is below the lower bound of background (noise) copy number for deletion or when the lower bound of the copy number ratio is above the upper bound of background (noise) copy number for amplification.

### Sanger Sequencing

Sanger sequencing was performed for detection of the Trp372Arg mutation in *YY1* gene. PCR was performed in 25 µl reactions. The reaction mixture for PCR consisted of the following reagents: 8% glycerol, 68 mM Tris-HCl with pH 8.3, 17 mM (NH_4_)_2_SO_4_, 0.01% Tween-20, 0.1 mg/ml BSA, 0.2 mM each dNTP, 1.5 units Taq polymerase, 0.12 pM each primer, 2.5 mM MgCl_2_. On top, 40-60 μl of mineral oil was layered. PCR reaction was performed with the following parameters: 95°C for 5 min, followed by 30 cycles of 95°C for 40 seconds, 61°C for 40 seconds and 72°C for 40 seconds. Final elongation was at 72°C for 5 minutes. Sanger sequencing was performed on ABI3500 according to the manufacturer’s protocol (Thermo Fisher, USA). Sequencing results were analyzed in Chromas software and compared to GenBank database using the BLAST algorithm. Primer sequences for Trp372Arg are listed in **Table 1**.

**Table 1:** Primer sequences for Trp372Arg and qPCR confirmations (genome assembly GRCh37/hg19).

Amplicon name	Forward primer	Reverse primer
Primers for Trp372Arg in YY1 gene		
Y1_372	GGGTCTGGTCAGAGTTGCTG	CCATCGAAGGGGCACACATA
Primers for qPCR		
B2M	TGCTGTCTCCATGTTTGATGTATCT	TCTCTGCTCCCCACCTCTAAGT
ATRXex1	TGTCGGCTTCTGTGATTGCT	TTTGAGCTGTGGGGAGGTTC
ATRXex9	CTTTCCCCGCCTGAGTCTTT	GGTGAGCAGGATGAGTCACA
CEBPA	ACAAACAAGGCTGAGGGTCC	GTGGGTCAGCTCTGAGGATG
IRS2ex1	GTTGAGGTAGTCCCCGTTGG	GAGGACAGTGGGTACATGCG
IRS2ex2	CGACAGCCCTCCAATCAAGT	ACCAGTGTGTGGCAGTTCTC
FOXL2	ACACACGTATTGGTCCGTCC	GTGCAGTCCATGGCTAGACG

### Quantitative PCR

Quantitative PCR (qPCR) is one of the methods that are widely used to detect copy number changes. This method is preferable due to its low consumable and instrumentation costs, high sensitivity and fast development time of assay (6–9). qPCR was performed to confirm the results of CNVPanelizer packages for *CEBPA* and *FOXL2* gene, the first and the second exons of *IRS2* gene and the first and the ninth exons of *ATRX* gene.

The qPCR mixes were prepared according to the GenPak PCR Core protocol (Isogene Lab ltd, Russia) in 20 µl reactions containing 1 ng genomic DNA and using SYBR green as a reporter and ROX as a reference dyes. All qPCR reactions were run in triplicate on a QuantStudio 5 Real-Time PCR System for Human Identification (Thermo Fisher Scientific, USA). The PCR conditions were as follows: 95°C for 5 min, followed by 40 cycles of 95°C for 20 seconds, 60°C for 20 seconds and 72°C for 20 seconds. Data were analyzed by the ΔΔCt method (10). The relative copy number was estimated by comparison with a normal blood control DNA sample. We used the *B2M* housekeeping gene as an endogenous control. Primer sequences for qPCR are shown in **Table 1**.

## Results

### Sanger Sequencing

Point mutation Trp372Arg (Chr14:100,743,807, hg19) in *YY1* gene was not found in the tested samples.

### Next Generation Sequencing

Median NGS read coverage was 486x, 461x, 471x, 454x for four tumor samples and 217x for a blood sample.

#### Single Nucleotide Variations

No pathogenic point mutations associated with any genetic syndrome including MEN1 were found in blood, neither pathogenic point mutations in tumor samples.

#### Copy Number Variations

##### CNVs in Tumors

**Figure 2:**
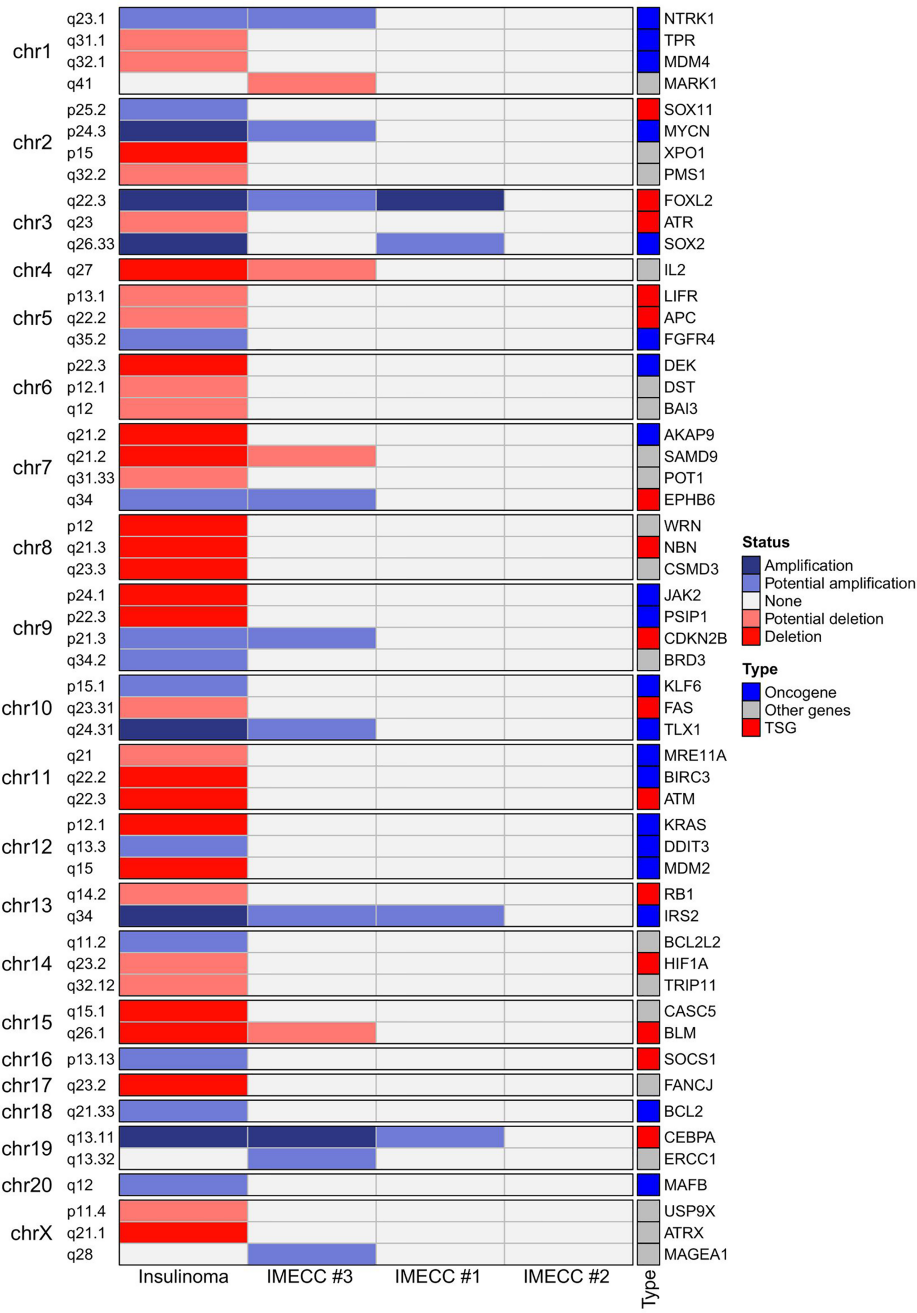
Heatmap of the results of CNVPanelizer.

CNVPanelizer in total has detected 54 genes within CNV regions, where 51, 14 and 4 genes were found in insulinoma, IMECCs #1 and #3, respectively (**Figure 2**). By using ONgene (11) and TSGgene (12) databases that aggregate information about oncogenes and tumor suppressor genes respectively, we identified several oncogenes and tumor suppressor genes with different CNV status in our tumor samples.

In insulinoma sample, ten oncogenes (*BCL2*, *DDIT3*, *FGFR4*, *IRS2*, *KLF6*, *MAFB*, *MYCN*, *NTRK1*, *SOX2* and *TLX1*) were with either putative or reliable status of gain, and nine tumor suppressor genes (*APC*, *ATM*, *ATR*, *BLM*, *FAS*, *HIF1A*, *LIFR*, *NBN* and *RB1*) were with putative or reliable status of loss.

With regards to IMECCs, in IMECC #1 sample two oncogenes, *SOX2* and *IRS2*, were with putative status of gain. In IMECC #3 four oncogenes, *IRS2*, *MYCN*, *NTRK1*, *TLX1*, were with putative status of gain and one tumor suppressor gene, *BLM*, with putative status of loss. In IMECC #2 according to CNVPanelizer no CNVs were detected.

**Figure 3:**
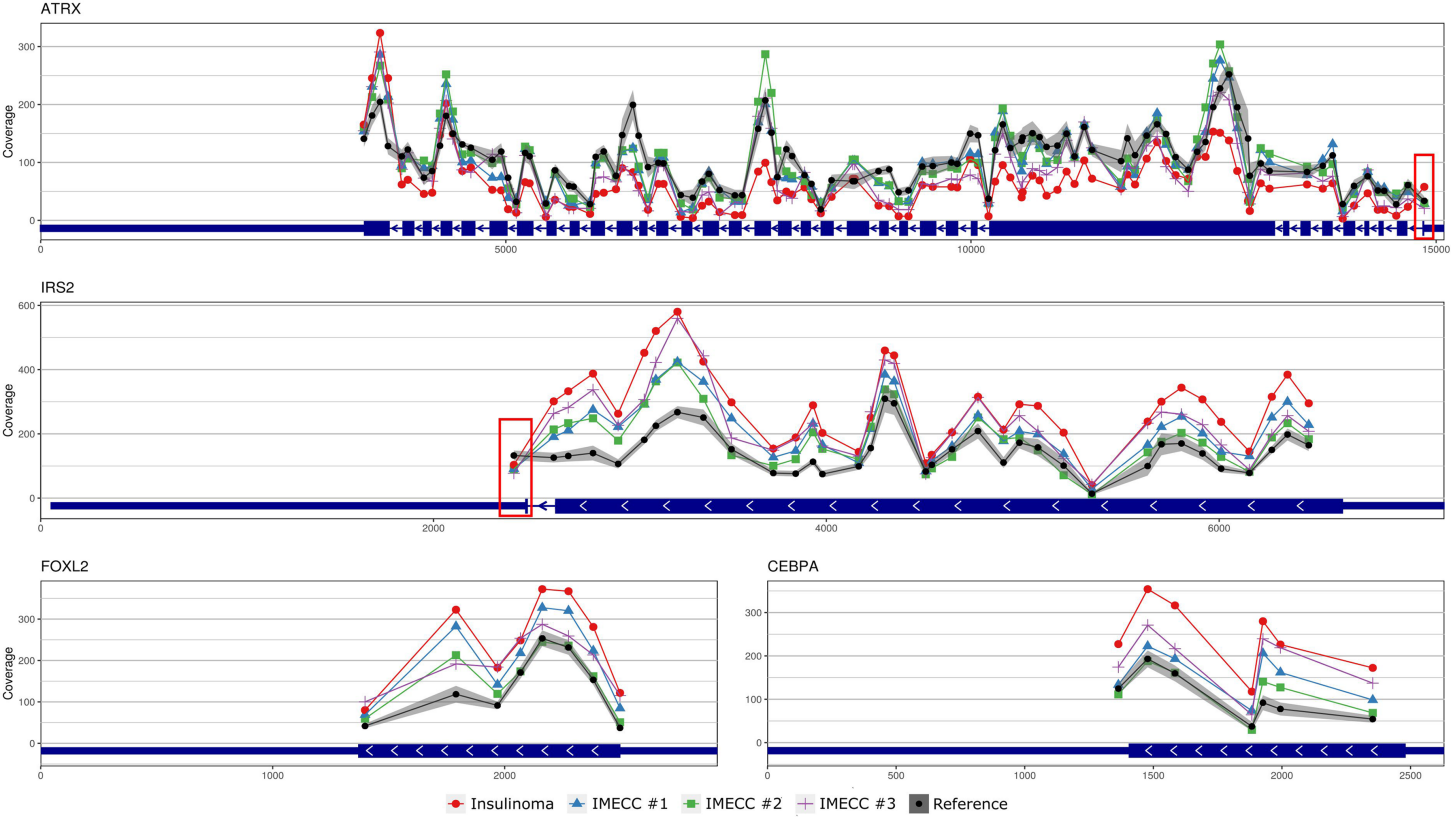
Visualization of CNVPanelizer results on genomic coordinates (genome assembly GRCh37/hg19) of the genes *ATRX*, *IRS2*, *FOXL2* and *CEBPA*.

With regards to the most common genes that mutate in pancreatic neuroendocrine neoplasms (panNENs) (*MEN1*, *ATRX* and *DAXX*) only loss of *ATRX* was detected in insulinoma sample. Visualizing the results of CNVPanelizer on genome coordinates (hg19) we have noticed that in the insulinoma sample the data for the first exon of *ATRX* was higher (shown in red rectangle) than the reference values, whereas the downstream exons were lower (**Figure 3**).

##### Recurrent CNVs

According to the results of CNVPanelizer, amplifications of *FOXL2*, *IRS2* and *CEBPA* genes were found in all samples except IMECC #2, (**Figure 2**). However, visualization of CNVPanelizer results (**Figure 3**) showed that in *IRS2* gene the second exon was lower in all tested samples including IMECC #2 compared to reference data, suggesting its loss.

### Quantitative PCR

Gain status was confirmed in all samples for *FOXL2* gene. For *CEBPA*, only in insulinoma and IMECC #1 gain status was confirmed (p-value <0.001 and <0.01 respectively).

Amplification of the first exon of the *IRS2* gene was also confirmed in all samples (p-value <0.001 for all samples). However, the data on the second exon was various. Results qPCR with CNVPanelizer matched only in insulinoma sample (p-value <0.01).

Amplification of the first exon of *ATRX* in insulinoma sample that we noticed on CNVPanelizer was confirmed with a statistical significance of p-value < 0.01. The downstream loss of *ATRX* which we checked in the ninth exon was also confirmed with statistical significance of p-value < 0.05. These results show that CNVPanelizer can reliably detect CNVs in separate exons.

## Discussion

In this study, we describe genetic alterations in tumors that refer to such rare phenomenon as insulinomatosis in a patient without known hereditary syndromes.

### Pancreatic Neuroendocrine Neoplasms

Pancreatic neuroendocrine neoplasms (panNENs or pNENs) are rare tumors of the pancreas that account for up to 2% of all pancreatic neoplasms. However, based on autopsy studies, prevalence of panNENs has been reported to be up to 10% (13). The 2017 World Health Organization classification divided panNENs into two categories, well-differentiated pancreatic neuroendocrine tumors (panNETs or PNETs) and poorly differentiated pancreatic neuroendocrine carcinomas (panNECs) (14). In the vast majority PNETs occur sporadically (~90%), but up to 5-10% are associated with genetic syndromes like multiple endocrine neoplasia type 1, neurofibromatosis type I, Von Hippel–Lindau syndrome and tuberous sclerosis complex (15). The most common functioning PNETs are insulinomas with an incidence of 4-7 per 100,000 persons per year (16–18). Most of insulinomas, more than 90%, are benign. Insulinomas are composed of producing beta cells that are actively secreting a large amount of insulin that results in episodic hyperinsulinemia and is the most frequent cause of persistent hyperinsulinemic hypoglycemia (1). In addition to Ki-67, which differentiates malignant and benign nature, tumor size is critical for survival rate prognosis. Thus, insulinomas >2 cm in diameter have a 10-year survival rate nearer to 30%, whereas for those <2 cm, the survival rate is close to 100% (19).

### Molecular Genetics of Tumors in Insulinomatosis

**Figure 4:**
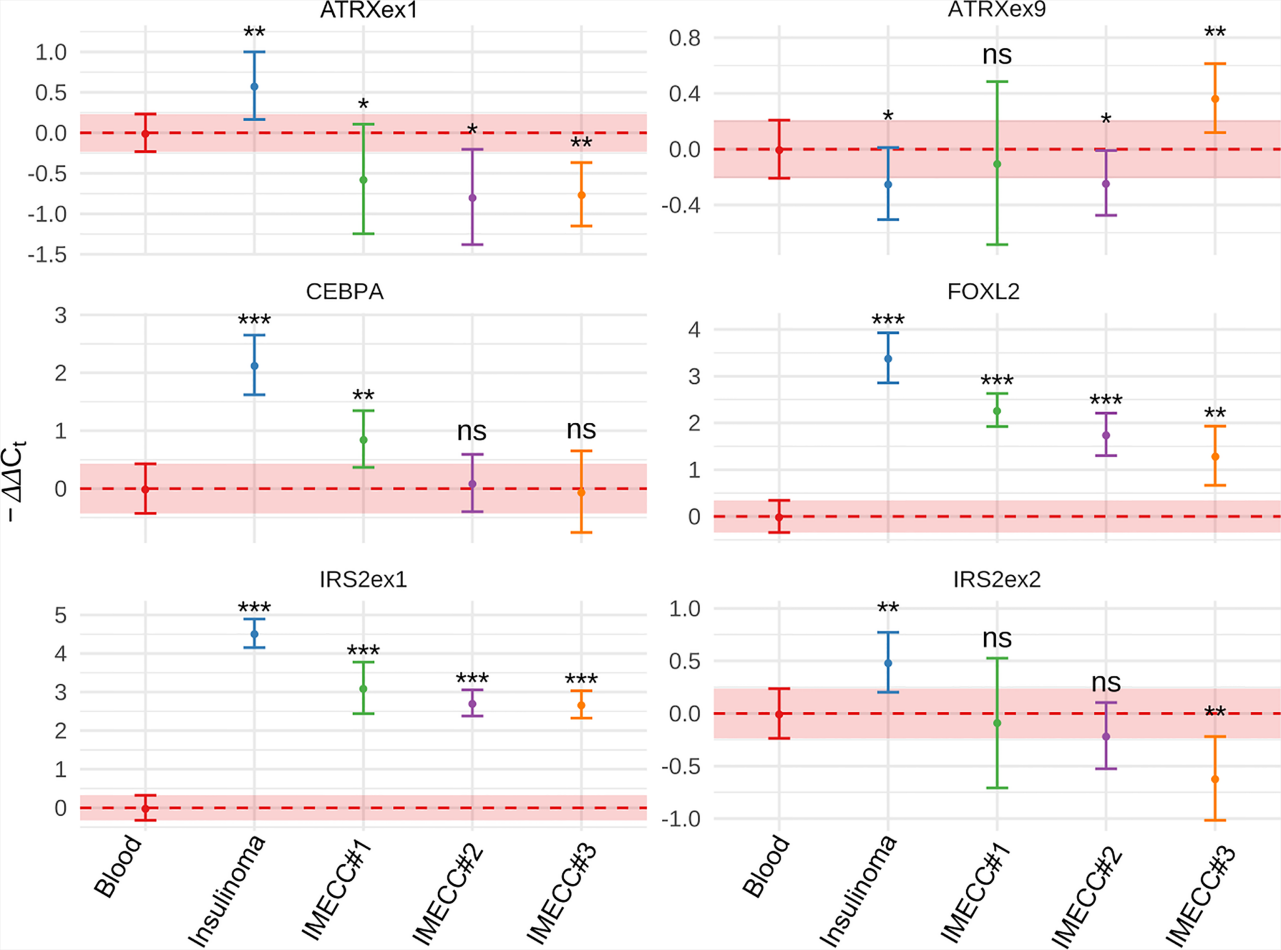
Results of qPCR in blood, insulinoma and IMECCs by using ΔΔCt method. *p-value < 0.05. **p-value < 0.01. ***p-value < 0.001. ns, not significant.

The molecular genetic of insulinomatosis is yet to be understood. This phenomenon is not related to mutation in *MEN1* gene and is more similar to sporadic benign insulinomas (1). In insulinomas, mutations in *MEN1*, *ATRX* and *DAXX* that are often mutated in all PNETs occur in no more than 10 percent: 3%, 8% and 3%, respectively (20). In addition many other genes were seen to be mutated in insulinomas (20, 21) and several regions with amplification (7p, 3p, 5q and 13q) were identified as early events and may be involved in tumorogenesis (22). However, the most common mutation in insulinoma is a gain of function mutation Trp372Arg in *YY1* gene that occurs in 30% in Asian population and in 13% in Caucasian (German) population (23, 24). Mutation Trp372Arg increases the activity of YY1 as transcription factor, which results in greater transcription of *IDH3A*, *UCP2* and increases the expression of *ADCY1* and *CACNA2D2* which regulate the insulin secretion (22). It is assumed that mutations in *YY1* gene are driver mutations for insulinomas (25). In our case of insulinoma, we did not find Trp372Arg mutation in the *YY1* gene nor mutations in *MEN1* or *DAXX*, however, loss of *ATRX* was found and confirmed by qPCR (**Figures 2**–**4**).

As for other CNVs, alterations were found in 13q34 (amplification of *IRS2*), 13q14.2 (loss of *RB1*) and 5q22.2 (loss of *APC*) (**Figures 2**, **3**). With regards to the precursor lesions of PNETs, the information about their molecular genetic profile is very limited. Microadenomas are shown to harbor mutations in *MEN1* (13, 14, 26, 27). Hadano et al. in their work showed that sporadic microadenomas have a significantly lower expression of ATRX and overexpression of cytokeratin-19 (CK19) compared to hyperplasia of pancreatic islet cells (13). In our studied samples of micro-tumors, we have not found alteration in *MEN1*, but gain status in *IRS2*, *FOXL2* and *CEBPA* genes was found, suggesting that they can play a role in the development of micro- to macro-tumors, to wit insulinomas, and, accordingly, insulinomatosis.

### Common Features and Filiation Between Micro- and Macro-Tumors

Besides the identity in monohormonality, our macro and micro-tumors samples share CNVs that harbor the same genes, *CEBPA*, *FOXL2* and *IRS2*. CCAAT enhancer-binding protein alpha (CEBPA) and Forkhead Box L2 (FOXL2*)* are known for being able to arrest or suppress cell proliferation respectively (28–30). The IRS2 (Insulin receptor substrate 2) protein plays an important role in the response to stimuli for cytokines and growth factors, like insulin and insulin-like growth factor 1, and promotes proliferation and survival of normal and cancer cells by mediating signaling from INSR, IGF1R, EPOR, MPL, VEGFR, LEP, GH and IFNB1/IFNG proteins. The stimulation of insulin receptor results in IRS2 association with the p85 subunit of PI3K and GRB2, activating the PI3K/AKT/MTOR and MAPK pathways and leading to proliferation and differentiation (31, 32).

It was shown that increased expression of CEBPA is involved in apoptosis of pancreatic β-cells exposed to proinflammatory cytokines IL1β, IFNγ, and TNFα (33). Also it was shown that CEBPA induce the transdifferentiation of B cells into macrophages, and in co-expression with the transcription factors Oct4 (Pou5f1), Sox2, Klf4 and Myc enhances reprogramming into induced pluripotent stem cells (34).

Mohanty in his work showed that overexpression of IRS2 stimulates proliferation of β-cells and increases insulin secretion. It also protects β-cells from d-glucose-induced apoptosis. On the contrary, the repression of IRS2 in INS-1 cells leads to downregulation of proliferation (35).

As insulinomatosis seems to be a disease that affects the entire population of β-cells, we can assume that this triad of genes, *CEBPA*, *FOXL2* and *IRS2*, participates in the disturbance of morphogenesis of β-cells, although of course, this statement requires more detailed investigations.

## Conclusion

Despite the fact that detection of cancer associated CNVs has traditionally been performed by microarray techniques, NGS-based bioinformatic methods are actively developing and becoming increasingly popular due to cost-efficiency. However, the NGS-based bioinformatics results still need to be confirmed with other molecular approaches. On the other hand, molecular approaches such as qPCR are sensitive to the quality of material, which can be challenging when the samples are from FFPE tissues.

Here we described a rare case of insulinomatosis with novel CNVs that are seen in multiple micro-tumors and a macro-tumor and harbor *CEBPA*, *FOXL2* and *IRS2* genes that can be involved in pancreatic neuroendocrine tumor pathogenesis, specifically insulinomatosis, and can provide new insights into the disturbance of morphogenesis of β-cells.

## Data Availability Statement

The datasets presented in this study can be found in online repositories. The names of the repository/repositories and accession number(s) can be found below: https://www.ncbi.nlm.nih.gov/, PRJNA752875.

## Ethics Statement

The studies involving human participants were reviewed and approved by Ethics Committee at the Research Centre for Medical Genetics, Moscow, Russia. The patients/participants provided their written informed consent to participate in this study. Written informed consent was obtained from the individual(s) for the publication of any potentially identifiable images or data included in this article.

## Author Contributions

IV, AS, and AV performed the surgical operation. LG performed the microscopy and immunohistochemistry. KA performed the DNA extraction, NGS, bioinformatics analysis, Sanger sequencing, and literature review. KK performed bioinformatics analysis and data visualization. LK and KA performed qPCR analysis. MS performed statistical analysis of qPCR results and assisted with bioinformatic analysis of CNV data. AE, SK, and VS administered the project. All authors contributed to the article and approved the submitted version.

## Funding

The research was carried out within the state assignment of Ministry of Science and Higher Education of the Russian Federation for RCMG. Statistical analysis in this work was supported by the Grant of the Government of the Russian Federation № 14.W03.31.0005.

## Conflict of Interest

The authors declare that the research was conducted in the absence of any commercial or financial relationships that could be construed as a potential conflict of interest.

## Publisher’s Note

All claims expressed in this article are solely those of the authors and do not necessarily represent those of their affiliated organizations, or those of the publisher, the editors and the reviewers. Any product that may be evaluated in this article, or claim that may be made by its manufacturer, is not guaranteed or endorsed by the publisher.
